# Re‐evaluating the prevalence and factors characteristic of catecholamine secreting head and neck paragangliomas

**DOI:** 10.1002/edm2.256

**Published:** 2021-06-02

**Authors:** Joshua D. Smith, Susan E. Ellsperman, Gregory J. Basura, Tobias Else

**Affiliations:** ^1^ Department of Otolaryngology – Head & Neck Surgery University of Michigan Medical School Ann Arbor MI USA; ^2^ Division of Metabolism, Endocrine, and Diabetes Department of Internal Medicine University of Michigan Medical School Ann Arbor MI USA

**Keywords:** adrenergic, catecholamines, functional, head and neck, metanephrines, paragangliomas, vasoactive

## Abstract

**Introduction:**

We sought to characterize the prevalence and factors characteristic of head and neck paragangliomas (HNPGLs) that secrete catecholamines to inform best practices for diagnosis and management.

**Methods:**

This was a retrospective cohort study from 2000 to 2020 at a single‐institution tertiary centre. One‐hundred fifty‐two patients (182 tumours) with HNPGLs with at least one measurement of urine or plasma catecholamines and/or catecholamine metabolite levels prior to treatment were included. We differentiated and characterized those patients with increased level(s) of any nature and those with ‘clinically significant’ versus ‘clinically insignificant’ catecholamine production.

**Results:**

Thirty‐one (20.4%) patients had increased catecholamine and/or catecholamine metabolite levels. In most patients, these levels were ≤5‐fold above the upper limit of the reference range. Four of these 31 patients with increased levels were ultimately found to have an additional catecholamine secreting mediastinal paraganglioma or pheochromocytoma. Fourteen of 31 patients with HNPGL were deemed clinically significant secretors of catecholamines based on hyper‐adrenergic symptoms and/or profound levels of normetanephrines. This cohort was enriched for patients with paragangliomas of the carotid body or cervical sympathetic chain and those with *SDHB* genetic mutations. Ultimately, the prevalence of clinically significant catecholamine secreting Hangs was determined to be 9.2% and 7.7% based on a per‐patient and per‐tumour basis, respectively.

**Conclusions:**

The rate of catecholamine excess in the current cohort of patients with HNPGLs was higher than previously reported. Neuroendocrine tumours of any anatomic subsite may secrete catecholamines, although not all increased laboratory level(s) are indicative of clinically significant catecholamine secretion causing symptoms or warranting adrenergic blockade.

## Graphical abstract

The prevalence of clinically significant catecholamine secreting head and neck paragangliomas was determined to be 7.7%–9.2% based on a per‐patient and per‐tumour basis, respectively. The rate of catecholamine excess in the current cohort of patients with HNPGLs was higher than previously reported. Neuroendocrine tumours of any anatomic subsite may secrete catecholamines, though not all increased laboratory level(s) are indicative of clinically significant catecholamine secretion causing symptoms or warranting adrenergic blockade.

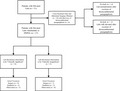

## 1 INTRODUCTION

Head and neck paragangliomas (HNPGLs) are rare tumours derived from paraganglial cells within autonomic ganglia of the carotid body (CBP), vagus nerve (VP), jugular bulb (JP), Jacobsen's nerve of the middle ear (TP) or cervical sympathetic chain (SCP).^1^ Due to their cell of origin, HNPGLs have the potential to actively synthesize and secrete catecholamines with potentially deleterious systemic effects. Patients with catecholamine secreting HNPGLs may present with symptoms of catecholamine excess, including sustained or intermittent hypertension and tachycardia, cardiac palpitations, diaphoresis and/or pallor. Regardless of symptomatology, failure to identify and treat catecholamine excess may cause significant morbidity and even mortality in these patients, particularly those undergoing surgery.^2^ As a result, contemporary clinical practice guidelines recommend biochemical testing of urine or plasma catecholamines and metabolites, usually metanephrine and normetanephrine levels, for all patients with newly diagnosed HNPGLs.^3^

The prevalence of catecholamine secreting HNPGLs is typically low (approximately 3%–4% of tumours).^4^, ^5^ However, this estimate is based primarily on data from limited series published 20 years ago. More recent evidence suggests that the rate of catecholamine secreting HNPGLs may exceed 10%.^6^ Recently, biochemical and genetic characteristics of HNPGLs, including standard treatment paradigms have been vastly transformed.^7^, ^8^ There now exists a clear gap in the literature regarding the true rate and factors characteristic of catecholamine secreting HNPGLs, particularly those whose functional status may pose significant challenges for peri‐operative management.

Here, we report a large series of patients with HNPGLs with the primary aim of characterizing the prevalence and features of HNPGLs that secrete catecholamines at a level significant enough to cause symptoms or warrant consideration of adrenergic blockade, herein termed ‘clinically significant’. In an era of increasingly personalized treatment for these tumours, our data may inform contemporary, best practices for biochemical screening and multi‐disciplinary management of HNPGLs.

## 2 METHODS

This was a retrospective analysis of a prospectively maintained clinical database of patients with HNPGLs presenting to our institution for evaluation and management between 2000 and 2020.^9^ Inclusion criteria for this study were as follows: (1) radiographically confirmed isolated or multi‐focal HNPGL; (2) previously untreated HNPGL tumour(s); and (3) at least one laboratory measurement of urine or plasma catecholamine or catecholamine metabolite levels prior to treatment onset.

Urine measurements included analysis of 24‐h excretion of fractionated normetanephrines and metanephrines, vaniyllmandelic acid (VMA), norepinephrine, epinephrine and dopamine via standard clinical assays.^10^ Similarly, plasma measurements included analysis of fractionated normetanephrines and metanephrines, VMA, norepinephrine, epinephrine and dopamine, as described.^10^ As expected, laboratory reference ranges were not uniform due to the twenty‐year study period, differences in clinical assays, and few patients with laboratories from other institutions that were not repeated upon presentation. As such, we recorded absolute laboratory levels and calculated a normalized ‘per cent of reference range’ level for each measurement as follows: [(absolute level—lower bound of reference range)/upper bound of reference range] x 100. Laboratory assessments were considered increased when the per cent of reference range level exceeded 100%.^11^

As previous authors have posited, it is a clear oversimplification to characterize HNPGLs as simply functional or not. Rather, HNPGLs exhibit a continuum of hormonal activity influencing clinical presentation and need for hemodynamic management.^12^, ^13^ Thus, the primary goal of this study was to investigate the rate and characteristics of ‘clinically significant’ catecholamine secreting HNPGLs. Clinically significant catecholamine secreting HNPGLs are defined as tumours in patients where (1) any laboratory level(s) are accompanied by clear hyper‐adrenergic symptoms at first presentation, as defined below; or (2) increased laboratory level(s) of normetanephrine ≥2‐fold were present with or without hyper‐adrenergic symptoms. We secondarily sought to determine rate and factors characteristic of ‘clinically insignificant’ catecholamine secreting HNPGL, defined as any patient with increased laboratory level(s) not meeting aforementioned criteria. Finally, we estimated the sensitivity and specificity of hyper‐adrenergic symptoms (defined below) at first presentation for predicting increased laboratory level(s) and clinically significant catecholamine secretion in our patient population.

Operational definitions for other recorded clinical variables are as follows: hyper‐adrenergic symptoms at first presentation were defined as explicit documentation of sustained or intermittent palpitations, tachycardia, diaphoresis, and/or tremors or new‐onset hypertension in conjunction with at least one of these other symptoms. Hypertension at first presentation was defined as systolic blood pressure ≥140 mmHg or diastolic blood pressure ≥90 mmHg. Tachycardia at first presentation was defined as resting heart rate >100 beats per minute.

Statistical comparisons between groups were made with chi‐square test and Student's *t* test for categorical and continuous variables, respectively. All statistical tests were two‐tailed and performed with SPSS Version 27 with a *p* ≤ .05 as the threshold for statistical significance. This study was deemed exempt from informed consent by the University of Michigan Institutional Review Board (IRB).

## 3 RESULTS

Our study cohort consisted of 280 patients with HNPGLs comprising 318 discrete tumours. There were significant differences evident among patients in whom laboratories were drawn/documented (*n* = 152, 182 tumours) versus not (*n* = 128, 136 tumours). Specifically, patients in the ‘labs drawn’ group were younger overall and more likely to endorse hyper‐adrenergic symptoms at presentation. HNPGL tumour subsite(s) also differed, with more JP and multi‐focal HNPGLs and fewer TP in the ‘labs drawn’ group overall (Table 1).

**TABLE 1:** Demographic, clinical and tumour characteristics of HNPGL patient cohorts

	Entire Cohort (*n* = 280)	Laboratories Not Drawn (*n* = 128)	Laboratories Drawn (*n* = 152)	*p* Value
Age, years	52.1 (13.7–85.2)	58.6 (16.8–85.2)	49.6 (13.7–82.3)	**<.01**
Sex				
Male	96 (34.3)	45 (35.2)	51 (33.6)	.78
Female	184 (65.7)	83 (64.8)	101 (66.4)	
Hyper‐adrenergic symptoms				
Present	30 (10.7)	5 (3.9)	25 (16.5)	**<.01**
Not Present	110 (39.3)	34 (26.6)	76 (50.0)	
Undocumented	140 (50.0)	89 (69.5)	51 (33.5)	
Tumor subsite				
CBP, Isolated	110 (39.3)	55 (43.0)	55 (36.2)	**<.01**
JP, Isolated	63 (22.5)	20 (15.6)	43 (28.3)	
TP, Isolated	30 (10.7)	23 (18.0)	7 (4.6)	
VP, Isolated	33 (11.8)	19 (14.8)	14 (9.2)	
SCP, Isolated	9 (3.2)	2 (1.6)	7 (4.6)	
Other HNPGL, Isolated	5 (1.8)	2 (1.6)	3 (2.0)	
Multi‐Focal HNPGL	30 (10.7)	7 (5.4)	23 (15.1)	
Disease category				
Benign	270 (96.4)	125 (97.7)	145 (95.4)	.31
Malignant	10 (3.6)	3 (2.3)	7 (4.6)	
Family history				
Positive	49 (17.5)	17 (13.3)	32 (21.1)	.09
Negative	231 (82.5)	111 (86.7)	120 (78.9)	
Succinate dehydrogenase (SDHx) mutation				
Positive	61 (21.8)	11 (8.6)	50 (32.9)	**<.01**
SDHA	3 (4.9)	0	3 (6.0)	
SDHB	19 (31.1)	4 (36.4)	15 (30.0)	
SDHC	7 (11.5)	2 (18.2)	5 (10.0)	
SDHD	29 (47.5)	5 (45.5)	24 (48.0)	
Other	3 (4.9)	0	3 (6.0)	
Negative	20 (7.1)	1 (0.8)	19 (12.5)	
No Testing	200 (71.5)	117 (91.4)	83 (54.6)	

Bolded values indicate significant p values (α = 0.05).

**FIGURE 1:**
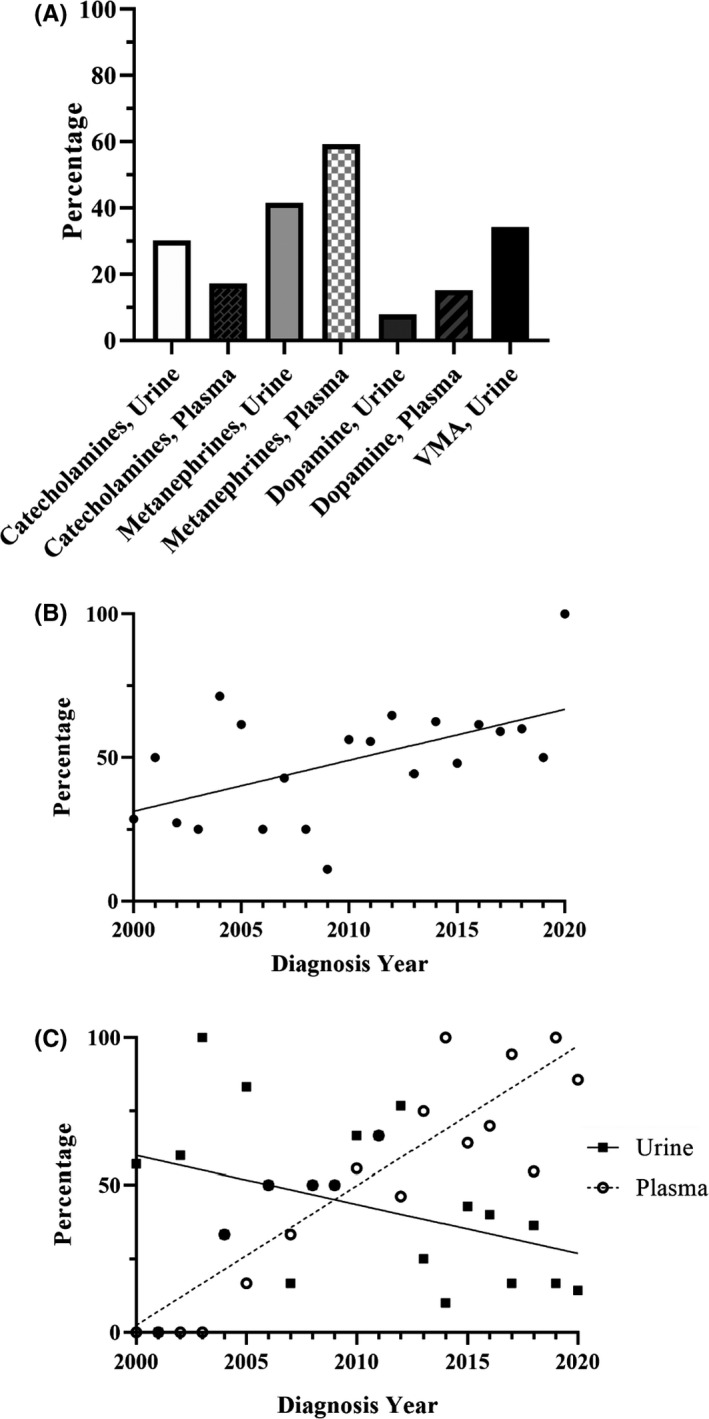
Percentage of patients (*n* = 152) who had specific laboratory assessments of catecholamine and catecholamine metabolite levels. ‘Catecholamines’ includes norepinephrine and epinephrine. ‘Metanephrines’ includes metanephrines and normetanephrines. (A). Trend in frequency of patients who had lab(s) drawn at first presentation (B). Significant but opposite trends in frequency of assessment of urine and plasma normetanephrines and metanephrines over time (C)

Within the ‘labs drawn’ group, the specific laboratory assessments of catecholamine and/or catecholamine metabolite levels varied considerably (Figure 1A). Twenty‐four‐hour urinary dopamine excretion was the least commonly employed test (*n* = 12, 7.9%) while plasma normetanephrines and metanephrines (*n* = 90, 59.2%) were most frequently assessed in our cohort. Over the course of the study period, we saw a modest increase in percentage of patients with HNPGL who had lab(s) drawn at first presentation (Figure 1B). Further, we saw a significant but opposite trend in the use of urine and plasma normetanephrine and metanephrine assessments over time (*p* < .01 for both trends, Figure 1C).

**FIGURE 2:**
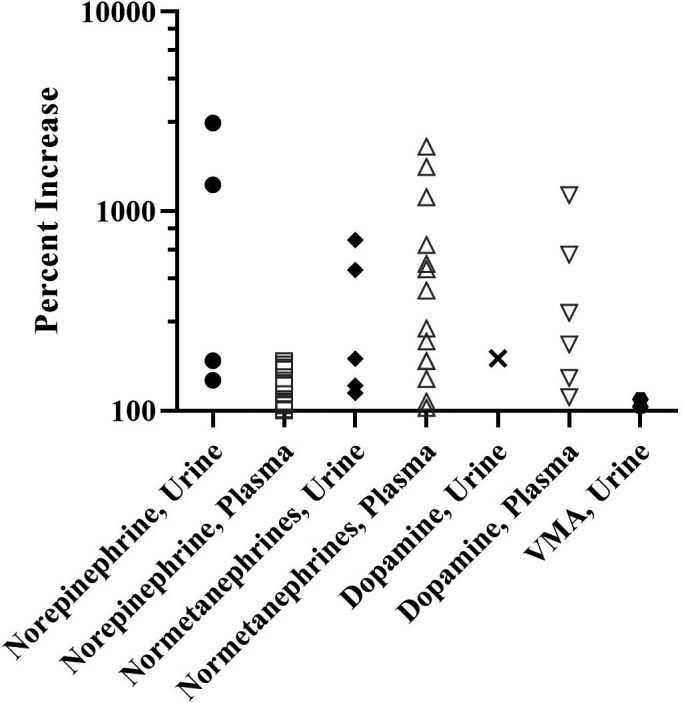
Per cent increase in specific laboratory levels in 31 of 152 (20.4%) patients. Each data point represents a discrete laboratory measurement, 45 in total. Note y axis is logarithmic

The median and interquartile range of all laboratory measurements are provided in Table S1. In total, 31 (20.4%) of 152 patients had one or more laboratory assessments showing increased catecholamine or catecholamine metabolite levels. In most patients with HNPGL, these levels were ≤5‐fold above the upper limit of the reference range, though a few individuals had profound laboratory levels of ≥10‐fold (Figure 2, Table S2).

**FIGURE 3:**
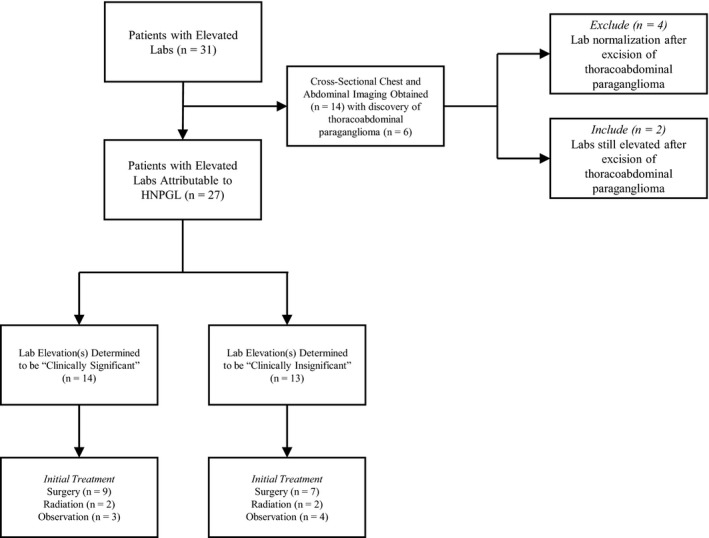
Flow diagram of source and clinical significance of catecholamine and/or catecholamine metabolite elevations in 31 HNPGL patients

The median (range) age of these 31 patients was 52.4 (18.3–78.2) years, and 22 (71%) were female. Roughly half of the 31 patients endorsed hyper‐adrenergic symptoms at first presentation, and all presented with benign HNPGLs of the following subsites: CBP (*n* = 10), JP (*n* = 9), TP (*n* = 2), VP (*n* = 2), SCP (*n* = 5) and multi‐focal HNPGL (*n* = 3). Detailed cohort characteristics of all 31 patients with HNPGL and increased laboratory levels are provided in Table S3. A flow diagram delineating the anatomic source and clinical significance (ie any laboratory level(s) accompanied by clear hyper‐adrenergic symptoms and/or laboratory level(s) of normetanephrine ≥2‐fold) of increased laboratory level(s) of catecholamines and/or catecholamine metabolites in these 31 patients is depicted in Figure 3. Notably, an additional catecholamine secreting mediastinal paraganglioma (MP) or adrenal pheochromocytoma was discovered in a sizable 19.4% (*n* = 6) of HNPGL patients with increased laboratory level(s).

Of the 13 HNPGL patients determined to have clinically insignificant increased laboratory level(s), none were treated with α‐ or β‐blockade prior to surgery or radiation or during observation. In those with clinically insignificant increases in laboratory level(s) who were treated surgically, review of anaesthesia records showed no instances of hemodynamic instability requiring pressors, aggressive fluid resuscitation or anti‐hypertensives.

In total, 14 patients in our cohort were determined to have evidence of clinically significant catecholamine secretion from their tumours (Table 2). This small cohort was particularly enriched for patients with tumours of the carotid body (CBP) and cervical sympathetic chain (SCP) as well as those with pathogenic *SDHB* mutations. Ten of these 14 patients were started on α‐ and/or β‐blockade after laboratory assessments were completed. We could not determine whether blockade was initiated in the remaining four patients due to insufficient clinical documentation or limited follow‐up duration. In summary, of 152 HNPGL patients with 182 total tumours, clinically significant catecholamine secretion was shown in 9.2% and 7.7% on a per‐patient and per‐tumour basis, respectively.

**TABLE 2:** Profile of HNPGL patients (*n* = 14) with clinically significant catecholamine secretion

Patient No.	Age, years	Sex	Hyper‐adrenergic symptoms	Hypertension and/or tachycardia	Tumor subsite(s)	SDHx mutation	Treatment
1	32	F	Yes	No	SCP	SDHC	Surgery
2	18	M	No	Yes	SCP	SDHB	Surgery
3	56	F	No	No	CBP	No Testing	Surgery
4	53	F	No	No	JP	No Testing	Surgery
5	55	F	No	Yes	JP	No Testing	Surgery
6	29	M	No	No	SCP	SDHB	Observation
7	44	F	No	Yes	JP	SDHB	Observation
8	53	M	Yes	Yes	CBP	SDHA	Surgery
9	50	F	Yes	Yes	CBP	Negative	Surgery
10	29	F	Yes	No	VP	SDHD	Radiation
11	40	F	Yes	No	CBP, MP	SDHB	Surgery
12	78	F	No	No	JP	SDHB	Radiation
13	25	M	No	No	SCP	SDHB	Surgery
14	69	F	Yes	No	CBP	SDHD	Observation

**FIGURE 4:**
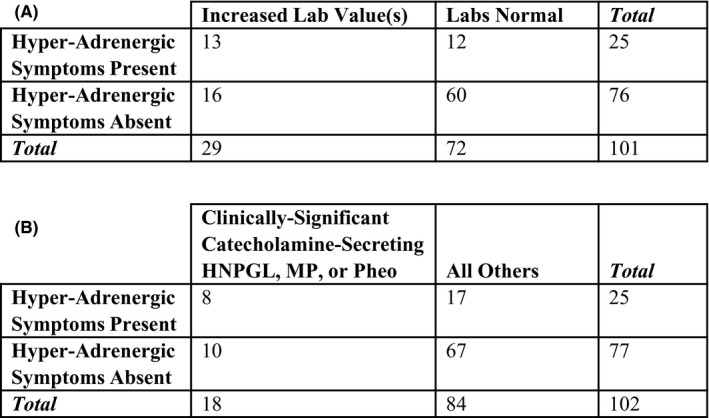
Contingency tables to calculate sensitivity and specificity of hyper‐adrenergic symptoms at first presentation for increased laboratory level(s) (A) and clinically significant catecholamine secretion from HNPGL, MP or pheochromocytoma (pheo) (B)

Lastly, we sought to evaluate the sensitivity and specificity of hyper‐adrenergic symptoms at first presentation for both increased laboratory level(s) and clinically significant catecholamine secretion. As expected, the sensitivity for both outcomes was quite low (44.8% and 44.4%, respectively). Conversely, specificity was much higher at 83.3% and 79.8%, respectively (Figure 4).

## 4 DISCUSSION

In recent years, diagnostic and treatment paradigms for HNPGLs have undergone vast change with the discovery of heritable succinate dehydrogenase (*SDHx*) mutations and a trend towards non‐surgical management.^14^ Traditionally, HNPGLs have been considered to rarely secrete catecholamines. This is in contrast to thoracoabdominal paragangliomas and adrenal pheochromocytomas derived primarily from sympathetic ganglia with comparatively higher rates of catecholamine hypersecretion.^15^ However, this assumption is based on limited series.^2^, ^4^ A contemporary re‐evaluation of the prevalence and features characteristic of catecholamine secreting HNPGLs in an era of evolving management is thus warranted.

While precise rates are poorly documented in the literature, it is evident that biochemical screening of newly diagnosed HNPGLs is not a uniform practice (Figure 1B).^9^ In our cohort, only 54.3% of patients had documented catecholamine screening, which reflects a twenty‐year study period and evolving testing recommendations and provider awareness. Patients who had catecholamine screening were younger and more likely to present with jugular or multi‐focal HNPGLs. Younger patients may have been more likely to consider surgical therapy versus watchful waiting thus prompting preoperative screening. Explicit documentation of assessment of hyper‐adrenergic symptoms occurred in only half of our patients. This could be due to incomplete documentation and limitations of retrospective data collection or a true inconsistency of assessing such symptoms on initial patient history. However, although not systematically analyzed, our experience shows that there is a significant fraction of asymptomatic patients despite significant increase in catecholamines. Either way, biochemical screening for newly diagnosed HNPGLs was also more common in those patients who endorsed such symptoms potentially attributable to hormonally active tumours.

As a rapid and easy screening tool, we hypothesized that more uniform assessment of hyper‐adrenergic symptoms at first presentation may be warranted to increase detection rates of catecholamine secreting HNPGLs. We found that sensitivity of such symptoms for any increased laboratory level(s) and clinically significant catecholamine secretion was quite low, at 44.8% and 44.4%, respectively. Specificity was moderately better, at 83.3% and 79.8%, respectively. These data support a few important conclusions. First, tumour secretion of catecholamines at levels that impact peri‐operative or long‐term management of HNPGLs may not always manifest with clear symptomatology. Second, objective measurement of catecholamine metabolite levels is imperative to determine whether newly diagnosed HNPGLs are indeed functional.

There are a number of plasma and urine laboratory tests available to the provider managing patients with HNPGLs. Evidence suggests these do not all hold equivalent sensitivity and specificity for diagnosing catecholamine secreting HNPGLs.^11^, ^16^ Based on an enhanced understanding of tumour catecholamine metabolism, the gold‐standard tests for diagnosis are plasma free or urinary fractionated metanephrines.^17^, ^18^ Assessment of plasma or urine catecholamines including norepinephrine, epinephrine, dopamine and VMA are associated with unacceptably high false‐positive and false‐negative rates.^3^ The precise tests ordered for our patients with HNPGL varied considerably through the study period, though we did see a statistically significant upward trend in the use of plasma free metanephrines over time (Figure 1C). At our institution, plasma free metanephrines have become the preferred screening test for HNPGLs due to superior test parameters, reliability of results and ease of specimen acquisition.

Due to vast heterogeneity in laboratory tests employed and our aim to delineate clinically significant versus clinically insignificant catecholamine secreting HNPGLs, we chose to first identify all patients in our cohort with increased laboratory level(s) of any kind. In 16 of these 31 patients, increased laboratory level(s) led providers to order cross‐sectional imaging of the chest and abdomen in search of another potentially functional tumour. Six of 31 patients (19.4%) were found to have a concomitant thoracoabdominal paraganglioma or adrenal pheochromocytoma (Figure 3). Due to the heritable nature and potential for multi‐focality, providers managing patients with incidental HNPGLs must be aware of the value of screening laboratories and strongly consider whole‐body, cross‐sectional imaging in those patients with evidence of catecholamine excess or familial paraganglioma predisposition syndromes.^19^

Based on our vast institutional experience, it is evident that increased laboratory level(s) indicative of catecholamine excess may not always lead to significant changes in clinical management. Thus, we sought to define and differentiate clinically significant versus clinically insignificant catecholamine secreting HNPGLs to better inform treatment/management decision‐making. Our operational definition for the former was based on reliability of plasma or urine metanephrine testing and/or the presence of unequivocal hyper‐adrenergic symptoms. When defined as such, 9.2% of HNPGL patients in our cohort had evidence of clinically significant catecholamine secretion. This stands in contrast to historically accepted rates, which estimated that only approximately 4% of HNPGLs were secretory.^4^ However, our observed rate is in line with a recent report by van Duinen et al,^6^ supporting more uniform attention to biochemical screening in patients with HNPGLs. Although the small number of patients with clinically insignificant elevations of catecholamines did not have any identifiable peri‐operative complications, it remains a matter of debate whether every patient with any catecholamine elevation should receive peri‐operative blockade.

Our data suggest that HNPGLs derived from both parasympathetic (eg CBP, JP, VP) and sympathetic (eg SCP) ganglia are capable of secreting catecholamines at a clinically meaningful level (Table 2). We found a 90% rate of heritable *SDHx* mutations in patients with clinically significant catecholamine secreting HNPGLs who underwent genetic testing. This supports strong consideration of genetic counselling in all such patients in parallel with their recommended treatment plan.^20^, ^21^ This will likely become more important as unique phenotype‐genotype relationships are further elucidated in an era of increasingly personalized diagnosis and therapy for these tumours.^22^ Due to the rarity of malignant HNPGL, it is interesting to speculate whether aggressive tumour behavior portends increased biochemical activity and catecholamine secretion. While we only had seven patients with malignant tumours and laboratory measurements at diagnosis, none of them had laboratory elevations indicative of catecholamine hypersecretion.

A recent paradigm shift, towards watchful waiting and/or radiation to avoid surgical morbidity for benign tumours was evident even in our patients with clinically significant catecholamine secreting HNPGLs. As such, biochemical screening for catecholamine excess has become essential for blood pressure and heart rate control with adrenergic blockade during the period of watchful waiting or radiation. For those patients treated surgically, our data reiterate the strong indication for biochemical screening in pre‐surgical workup to avoid rare but catastrophic peri‐operative complications.^23^

A measurable percentage (8.6%) of our cohort had clinically insignificant catecholamine secretion as we defined it. These patients had increased laboratory levels that were irreproducible, non‐specific or minor and not associated with hyper‐adrenergic symptoms. When patients first present to an otolaryngologist—head and neck surgeon, consultation with an endocrinologist with expertise in such tumours is ideal to help interpret laboratory levels and advise on appropriate next steps in diagnosis and management. While initiation of adrenergic blockade may not be immediately required for these individuals, the questionable catecholamine excess may prompt additional testing, imaging or genetic screening.

## 5 CONCLUSIONS

The rate of catecholamine excess in patients with HNPGLs may be higher than previously thought. Tumours of any anatomic subsite may secrete catecholamines, although not all increased laboratory level(s) are indicative of clinically significant catecholamine secretion causing symptoms or warranting adrenergic blockade. Our series provides a comprehensive, contemporary update on biochemical profiles of HNPGL in an era of evolving diagnostic and management standards for these tumours.

## CONFLICT OF INTEREST

The authors have no conflicts of interest to disclose relevant to this manuscript.

## AUTHOR CONTRIBUTION

We certify that all authors have met the following criteria for authorship: (1) Have made substantial contributions to conception, design, acquisition and analysis of data. (2) Were involved in drafting and revising the manuscript. (3) Have given final approval of the version to be published and take full responsibility for its content. (4) Agree to be accountable for all aspects of the work.

## Supporting information

Table S1

Table S2

Table S3

## Data Availability

Data are available on request from the authors.
