# Supplementary material for: Re‐evaluating the prevalence and factors characteristic of catecholamine secreting head and neck paragangliomas
Source: Endocrinol Diabetes Metab. 2021 Jun 2;4(3):e00256. doi: 10.1002/edm2.256 (PMC8279627; doi:10.1002/edm2.256)
Supplement: Supplementary file 1 — Table S1 [file EDM2-4-e00256-s002.docx]

|  | Number of Measurements | Median (IQR) Measurement, Absolute Values | Median (IQR) Measurement, Percent of Reference Range |
| --- | --- | --- | --- |
| Urine Measurements |  |  |  |
| Norepinephrine, μg/24 hr | 46 | 41.0 (26.6 – 57.3) | 41.0 (21.1 – 55.3) |
| Epinephrine, μg/24 hr | 45 | 8.2 (4.8 – 11.1) | 40.3 (23.5 – 55.5) |
| Normetanephrines, μg/24 hr | 63 | 303 (209 – 462) | 38.9 (22.2 – 67.6) |
| Metanephrines, μg/24 hr | 62 | 116 (79.8 – 137.5) | 37.7 (26.1 – 46.6) |
| Dopamine, μg/24 hr | 12 | 215.5 (155.4 – 264.8) | 50.2 (27.0 – 58.2) |
| VMA, mg/24 hr | 52 | 4.75 (3.6 – 6.0) | 52.7 (37.7 – 63.5) |
| Plasma Measurements |  |  |  |
| Norepinephrine, pg/mL | 26 | 521 (357.8 – 714.5) | 92.9 (59.6 – 113.5) |
| Epinephrine, pg/mL | 24 | 49 (22.5 – 65.5) | 43.0 (21.8 – 65.5) |
| Normetanephrines, nmol/L | 90 | 0.59 (0.40 – 0.73) | 65.0 (44.4 – 80.8) |
| Metanephrines, nmol/L | 90 | 0.20 (0.20 – 0.22) | 40.0 (40.0 – 44.0) |
| Dopamine, pg/mL | 23 | 58 (30 – 77) | 70.0 (46.0 – 117.0) |
