# Supplementary material for: Re‐evaluating the prevalence and factors characteristic of catecholamine secreting head and neck paragangliomas
Source: Endocrinol Diabetes Metab. 2021 Jun 2;4(3):e00256. doi: 10.1002/edm2.256 (PMC8279627; doi:10.1002/edm2.256)
Supplement: Supplementary file 2 — Table S2 [file EDM2-4-e00256-s003.docx]

| Patient No. | Norepinephrine, Urine (μg/24 hr) | Norepinephrine, Plasma (pg/mL) | Normetanephrine, Urine (μg/24 hr) | Normetanephrine, Plasma (nmol/L) | Dopamine, Urine (μg/24 hr) | Dopamine, Plasma (pg/mL) | VMA, Urine (μg/24 hr) |
| --- | --- | --- | --- | --- | --- | --- | --- |
| 1 | 3735 (2766) |  | 3080 (717) |  |  |  |  |
| 2 |  |  |  | 19 (2111) |  |  |  |
| 3 |  | 548 (110) |  |  |  | 605 (605) |  |
| 4 |  | 851 (170) |  | 0.93 (103) |  |  |  |
| 5 |  | 822 (164) |  |  |  | 117 (117) |  |
| 6 |  | 604 (121) |  | 1.3 (144) |  |  |  |
| 7 |  |  | 972 (123) | 4.9 (441) |  |  |  |
| 8 |  | 685 (137) |  |  |  |  |  |
| 9 | 131 (179) |  | 3442 (382) | 1.6 (178) | 677 (183) |  |  |
| 10 |  |  | 352 (116) |  |  |  |  |
| 11 | 1355 (1355) |  | 4679 (617) | 15 (1667) |  |  |  |
| 12 |  |  |  | 4.6 (511) |  |  |  |
| 13 |  |  |  | 2.0 (222) |  |  |  |
| 14 |  |  |  | 3.6 (400) |  |  |  |
| 15 |  |  |  |  |  |  | 10.5 (105) |
| 16 |  |  |  |  |  | 146 (146) |  |
| 17 |  | 883 (177) |  |  |  |  |  |
| 18 |  | 509 (102) |  |  |  |  |  |
| 19 |  |  |  |  |  | 241 (1205) |  |
| 20 |  | 533 (107) |  |  |  |  |  |
| 21 | 94 (142) |  | 736 (182) | 1.0 (100) |  |  |  |
| 22 |  |  |  |  |  | 62 (310) |  |
| 23 |  | 803 (161) |  |  |  |  |  |
| 24 |  | 550 (110) |  |  |  |  |  |
| 25 |  |  |  |  |  |  | 11.4 (114) |
| 26 |  | 534 (107) |  |  |  |  |  |
| 27 |  |  |  |  |  | 43 (215) |  |
| 28 |  |  | 3861 (508) | 10.6 (1178) |  |  |  |
| 29 |  |  |  | 6.1 (678) |  |  |  |
| 30 |  |  |  | 1.6 (178) |  |  |  |
| 31 |  |  |  | 2.33 (259) |  |  |  |
