# Supplementary material for: Re‐evaluating the prevalence and factors characteristic of catecholamine secreting head and neck paragangliomas
Source: Endocrinol Diabetes Metab. 2021 Jun 2;4(3):e00256. doi: 10.1002/edm2.256 (PMC8279627; doi:10.1002/edm2.256)
Supplement: Supplementary file 3 — Table S3 [file EDM2-4-e00256-s001.docx]

|  | Labs Elevated  (n = 31) |
| --- | --- |
| *Age, y* | 52.4 (18.3 – 78.2) |
| *Sex* |  |
| Male | 9 (29.0) |
| Female | 22 (71.0) |
| *Hyper-Adrenergic Symptoms* |  |
| Present | 13 (41.9) |
| Not Present | 16 (51.6) |
| Undocumented | 2 (6.5) |
| *Tumor Subsite* |  |
| CBP, Isolated | 10 (32.3) |
| JP, Isolated | 9 (29.0) |
| TP, Isolated | 2 (6.5) |
| VP, Isolated | 2 (6.5) |
| SCP, Isolated | 5 (16.1) |
| Multi-Focal HNPGL | 3 (9.6) |
| *Succinate Dehydrogenase (SDHx) Mutation* |  |
| SDHA | 1 (3.2) |
| SDHB | 7 (22.6) |
| SDHC | 2 (6.5) |
| SDHD | 2 (6.5) |
| Negative | 4 (12.8) |
| No Testing | 15 (48.4) |
| *Initial Treatment* |  |
| Surgery | 19 (61.3) |
| Radiation | 4 (12.8) |
| Observation | 8 (25.8) |
